# A Quality Improvement Project to Implement Choking Prevention and First Aid Education in Prader–Willi Syndrome Caregivers

**DOI:** 10.3390/jcm10214993

**Published:** 2021-10-27

**Authors:** Kathryn S. Obrynba, Kathryn Anglin, Amy Moffett, Tracie Steinke, Manmohan K. Kamboj

**Affiliations:** 1 Division of Endocrinology and Diabetes, Nationwide Children’s Hospital, Columbus, OH 43205, USA; kathryn.anglin@nationwidechildrens.org (K.A.); amy.moffett@nationwidechildrens.org (A.M.); Tracie.steinke@nationwidechildrens.org (T.S.); manmohan.kamboj@nationwidechildrens.org (M.K.K.); 2 Department of Pediatrics, The Ohio State University, Columbus, OH 43205, USA; 3 Department of Clinical Nutrition and Lactation, Nationwide Children’s Hospital, Columbus, OH 43205, USA

**Keywords:** Prader–Willi syndrome, quality improvement, choking

## Abstract

Prader–Willi syndrome (PWS) is a complex neurodevelopmental genetic disorder characterized by hypotonia and hyperphagia. Consequently, individuals with PWS are at high risk of choking, and choking is a leading cause of morbidity and mortality. The aim of this quality improvement (QI) project is to provide choking prevention and first aid education from 0% to 80% of PWS caregivers seen in a multidisciplinary PWS clinic, and to assess the effectiveness of this education program. A QI initiative was developed to standardize and implement choking prevention and first aid education for PWS caregivers. Using a Likert scale, pre- and post-education assessments were conducted to measure caregiver (1) awareness of the PWS choking risk, (2) self-reported knowledge of choking prevention strategies, and (3) comfort in providing choking first aid. The American Heart Association Family and Friends^®^ CPR (Dallas, TX, USA) curriculum was utilized. Education was provided during a regularly scheduled PWS clinic appointment. At project conclusion, 45/52 (87%) of PWS caregivers received education. A post-education assessment revealed an improvement in PWS caregivers’ awareness of choking risk, self-reported knowledge of choking prevention strategies, and comfort in providing choking first aid. This QI project supports a practice change to implement choking prevention and first aid education as standard process within our PWS clinic.

## 1. Introduction

Prader–Willi syndrome (PWS) is a complex neurodevelopmental genetic disorder with cardinal features of hypotonia and hyperphagia. This, in turn, leads to poor oral coordination and a decreased gag reflex [1]. As a result, PWS individuals are at an increased risk for choking compared to the typical population and choking is reported as the cause of death in 6–8% [2,3]. Despite this information, there may be an unawareness within the PWS community of choking risk.

In order to prevent choking in PWS individuals, it is important for caregivers to provide a food-secure environment, avoid high-risk foods, supervise meals, and know how to perform first aid in the event of choking. We, therefore, seek to standardize and implement choking prevention and first aid education to PWS individuals and their caregivers. The specific aim of this quality improvement (QI) study is to implement a choking prevention and first aid education program from 0% to 80% of PWS caregivers, and to assess the effectiveness of the education by evaluating the change in caregiver awareness of choking risk in PWS individuals, self-reported knowledge of choking prevention strategies, and comfort in providing choking first aid.

## 2. Materials and Methods

### 2.1. Setting

The PWS clinic at Nationwide Children’s Hospital in Columbus, Ohio, followed over 50 children and young adults with PWS with care coordinated through the section of endocrinology. PWS patients and caregivers returned to the clinic generally every 3–4 months, while some patients and caregivers who live further away returned to the clinic every 6–12 months. The multi-disciplinary team consisted of pediatric endocrinologists, geneticist and genetic counselor, neurologist, psychologist and psychometrician, registered dietitian, social worker, pediatric nurse practitioner, endocrinology nurses, and a nurse coordinator. Before the initiation of this project, there was no standardized approach to discuss choking risk, reviewing choking prevention strategies, or providing choking first aid education. We included all caregivers of individuals with PWS seen in the PWS clinic during the intervention period. All PWS caregivers were offered the interventions/education regardless of prior knowledge or training in choking first aid.

### 2.2. Interventions

A QI team was formed for the purpose of implementing choking first aid education into our routine clinic practice. The team included endocrinologists, nurse practitioners, endocrinology nurses, and a nurse coordinator. Following the Institute for Healthcare Improvement model, the QI team utilized key driver diagrams and effort–impact prioritization to help guide efforts for interventions to maximize improvement. A Certified Professional Healthcare Quality/Certified Lean Six Sigma Black Belt coached our project team.

**Figure 1:**
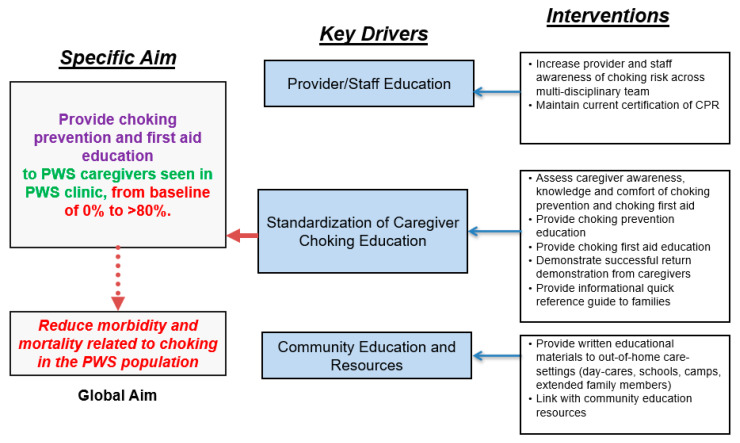
Key driver diagram summarizing the aim, key drivers, and interventions completed during the quality improvement study.

Project team members identified several key drivers: (1) provider and staff education, (2) standardization of caregiver choking education, (3) community education (Figure 1).

Interventions targeting each key driver were identified and tested using Plan–Do–Study–Act (PDSA) cycles to analyze the success of the interventions for full implementation versus development of another change cycle over an 18-month period [4]. The project team conducted a chart review prior to each clinic to determine which PWS caregivers had not received choking prevention and first aid education. Key interventions are summarized below.

#### 2.2.1. Provider/Staff Education

The QI team discussed choking risk in PWS individuals with the entire PWS multidisciplinary team at project initiation and on an ongoing basis to increase awareness and stress importance of this project. QI team members involved in this project were previously certified in cardiopulmonary resuscitation (CPR) and were encouraged to maintain their CPR certification for the duration of the project.

#### 2.2.2. Standardization of Caregiver Choking Education

- Pre-education Assessment.

On arrival to a regularly scheduled PWS clinic visit, caregivers completed a pre-education survey to assess their awareness of choking risk in individuals with PWS, their self-reported knowledge of choking prevention strategies, and their comfort in providing choking first aid (Supplementary File S1). Surveys were administered on paper and completed by the caregiver in a private setting. Answers were collected using a 5-point Likert scale with “1” being none/not at all aware/knowledgeable/comfortable and “5” being very aware/knowledgeable/comfortable. Education and materials were provided in English, and an interpreter was used for caregivers who were non-English speaking.

b. Caregiver choking education and training.

Following completion of the pre-education survey, caregivers were directed into a private teaching room within the clinic space to meet with an endocrinology nurse or nurse practitioner serving as the education facilitator. Caregivers were first informed of increased choking risk in PWS and were provided verbal and written strategies to help prevent choking episodes at home. This included encouragement of supervised meals, careful food portioning and preparation, and diet modification to avoid high-risk choking foods. Information provided was readily available through the Prader–Willi syndrome Association USA© website [5]. Caregivers watched a five minute video containing the choking portion of The American Heart Association Family and Friends^®^ Cardiopulmonary Resuscitation (CPR) curriculum [6]. Either the infant, child, or adult curriculum was used dependent on the age and/or size of the PWS individual. Caregivers demonstrated choking first aid (Heimlich) maneuvers on either infant, child, or adult CPR training mannequins. Completion of caregiver choking prevention and first aid education was documented in the electronic medical record for tracking purposes. Caregivers did not receive formal CPR certification. The PWS individual was encouraged to participate in the education session as maturity and understanding allowed, although education was targeted primarily towards the caregiver.

c. Post-education Assessment.

Immediately following education, caregivers completed an identical post-education survey to reassess their awareness of choking risk in individuals with PWS, their self-reported knowledge of choking prevention strategies, and their comfort in providing choking first aid. Although not assessed systematically, general observations from the education facilitator and unsolicited feedback from PWS caregivers regarding the education program were collected via written documentation and verbal recall.

#### 2.2.3. Community Education and Resources

Caregivers were provided written educational materials on choking prevention and first aid to distribute to out-of-home care settings such as day cares, schools, camps, and extended family members. As caregivers did not receive formal CPR certification during their education, they were provided with information to sign up for a local, full CPR certification course.

### 2.3. Measures and Analysis

#### 2.3.1. Process of Providing Choking First Aid Education

Our primary outcome measure was the percentage of PWS caregivers who received choking prevention and first aid education. Data were reviewed monthly with the QI team and were plotted on a control chart.

#### 2.3.2. Choking Awareness, Self-Reported Knowledge, and Comfort in Providing Choking First Aid

Our secondary outcome measure was the change in PWS caregiver awareness of choking risk in PWS individuals, self-reported knowledge of choking prevention strategies, and comfort in providing choking first aid using pre- and post-education assessment Likert scores. Data were reviewed at the completion of the QI initiative.

### 2.4. Statistics

Statistical analysis was performed using Systat statistical software (version 13 Systat Software Inc., San Jose, CA, USA). The Shapiro–Wilk test was used to assess the parametric distribution of the variables studied. The Wilcoxon Signed rank test was used to assess for differences between paired pre and post-test data with a *p*-value < 0.05 to indicate statistical differences. All data are expressed as median (25–75% CI).

### 2.5. Ethical Considerations

This QI work involved the implementation of best practices into our PWS clinic to optimize routine care. As such, it was identified as QI and did not involve human subjects research. Therefore, Institutional Review Board and approval were not required.

## 3. Results

**Figure 2:**
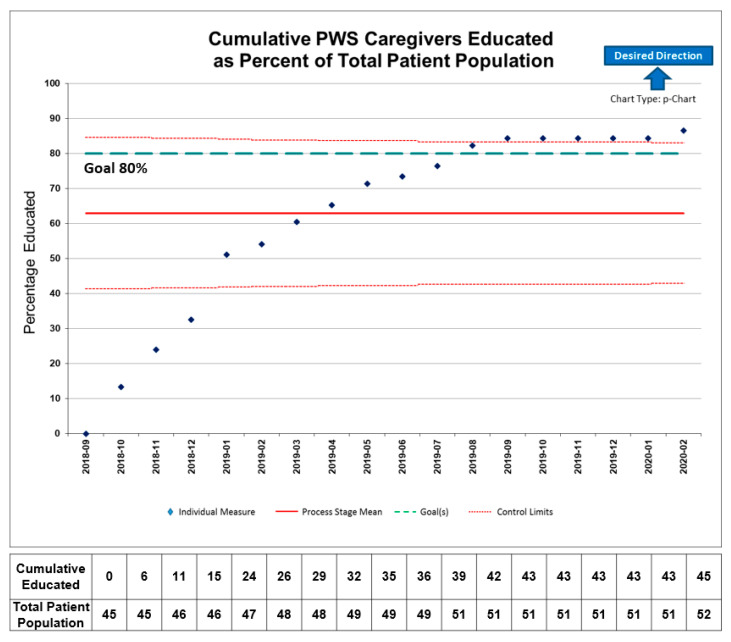
Cumulative PWS caregivers who received choking prevention and first aid education as percent of total population.

During the 18-month intervention period, 52 PWS caregivers were offered choking prevention and first aid education. A total of 45 (87%) PWS caregivers completed the education session and pre- and post-education surveys (Figure 2). Demographic data for the PWS individuals and their caregivers are listed in (Table 1).

### 3.1. Awareness of Choking Risk in PWS Individuals

**Figure 3:**
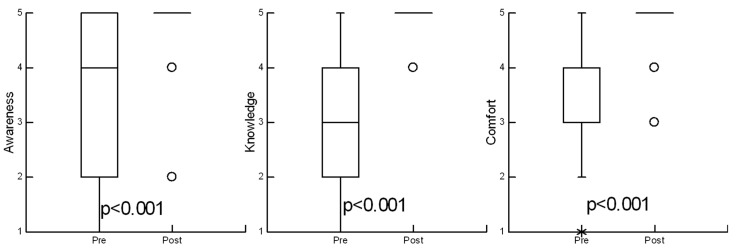
Median score pre and post-choking education. Boxes are median and 25–75th percentiles. Whiskers represent upper and lower fences 1.5× interquartile range. * points inside outer fences 3× interquartile range. Open circles are extreme outliers.

Prior to the choking prevention and first aid education, 12 PWS caregivers (26%) reported no to little prior awareness that individuals with PWS are at increased risk for choking. Following education, the majority of PWS caregivers 44 (97%) reported they were moderately to very aware of this choking risk. The median Likert score for PWS caregiver awareness of choking risk increased from four [2, 5] to five [5, 5] from the pre-education to post-education assessment (*p* < 0.001) (Figure 3).

### 3.2. Self-Reported Knowledge of Choking Prevention Strategies

Prior to the choking prevention and first aid education, 12 (35%) of PWS caregivers reported no to little knowledge of choking prevention strategies. Following education, 45 (100%) of PWS caregivers reported they were moderately to very knowledgeable on ways to prevent choking in PWS individuals. The median Likert score for knowledge of choking prevention strategies increased from three [2, 4] to five [5, 5] from the pre-education to post-education assessment (*p* < 0.002) (Figure 3).

### 3.3. Comfort in Providing Choking First Aid

Prior to the choking prevention and first aid education, 11 (24%) of PWS caregivers reported little to no comfort in providing choking first aid. Following education, no caregivers reported feeling uncomfortable providing choking first aid, and 43 (95%) of caregivers reported they were moderately to very comfortable in providing choking first aid. The median Likert score for comfort in providing choking first aid increased from four [3, 4] to five [5, 5] from the pre-education to post-education assessment (*p* < 0.001) (Figure 3).

### 3.4. Feedback from PWS Caregivers

The amount of time required for each education session was approximately 10–15 min, which was viewed favorably by the caregivers and families. Feedback obtained at the time of conducting the post-education assessment was generally positive, with comments in general attesting to the usefulness of the education. Observations and comments from PWS caregivers included the following:

One mother, who was already certified in CPR, commented “it was very helpful to specifically concentrate on choking prevention and choking first aid, since this is a smaller segment of the entire CPR certification course”.

A father, who was a physician, commented: “I found the resources and training helpful as it was specifically tailored towards children with PWS.”

In general, PWS caregivers appreciated the opportunity to include PWS individuals in the education as maturity and understanding allowed, and especially for the opportunity to teach the PWS individual how to make the universal sign for choking.

## 4. Discussion

This QI initiative successfully reported the implementation and assessment of a standardized choking prevention and first aid education program for PWS caregivers within our multi-disciplinary PWS clinic. At the study’s conclusion, the majority of PWS caregivers (87%) seen in the clinic over an 18-month intervention period received choking prevention and first aid education. A review of pre- and post-education assessments revealed an improvement across all measures, including caregivers’ awareness of PWS choking risk, self-reported knowledge of choking prevention strategies, and comfort in providing choking first aid.

Choking is a leading cause of morbidity and mortality among children in the United States, with many choking episodes being related to food [7]. Children with chewing and swallowing disorders, including children with PWS, are at a greatest risk for food related choking. Although the risk for a serious choking episode is generally greatest under the age of 5 years in typically developing children, the risk for a serious choking episode persists throughout life in the PWS population. Various surveys on the PWS population report a history of choking in 18–34% of PWS individuals, with 50% of choking episodes occurring at age 6 years or older [1,8,9]. However, discrepancies regarding the severity of choking episodes are noted, with as few as 5.9% to as many as 40% of choking incidents reported severe enough to require the Heimlich maneuver [7,8]. Regardless, choking has been reported as the cause of death in up to 8% of PWS individuals, including in children less than the age of 8 years old [1].

We found that prior awareness of choking risk in PWS was surprisingly low in PWS caregivers, with a quarter of caregivers having no to little prior knowledge of this risk. In addition, over one third of caregivers reported no to little knowledge of choking prevention strategies. Following our choking education, all caregivers reported some degree of awareness of choking risk and all reported improved knowledge on strategies to prevent choking. Given the PWS population is at such a great risk for morbidity and mortality related to choking, it is imperative that PWS caregivers are well informed and are provided education on choking prevention techniques and choking first aid maneuvers.

We realized that establishing a choking prevention and first aid education program was cost effective and did not pose a significant burden or workload to our clinic or organization. Many resources, including those published by the Prader–Willi syndrome Association USA© website (Sarasota, FL, USA) [3], were found readily on-line at no cost to our clinic. Our clinic purchased The American Heart Association Family and Friends^®^ Cardiopulmonary Resuscitation (CPR) curriculum [4] and CPR training mannequins online at a negligible cost. Choking prevention and first aid education are now implemented as part of our routine clinic workflow in our multi-disciplinary PWS clinic. As new patients and families have subsequently joined our clinic population, we provide choking prevention and first aid education at new patient visits. In addition, a discussion of choking risk is now routinely discussed with caregivers on an ongoing basis and is part of a routine discussion and counseling from our registered dietitian. We take the opportunity to renew this conversation, especially around the holidays when there are more likely to be large holiday gatherings with an abundance of food, during which time the risk for choking is greatest.

Similar to PWS, the Down syndrome population may also be at risk for swallowing dysfunction and choking [10]. A survey of caregivers of adults with learning disabilities reported that 42% of individuals had one or more choking episode, with Down syndrome being higher risk [11]. Despite these observations in the Down syndrome population, such as PWS, caregiver awareness of this risk may also be low. Likewise, any caregiver of an individual with a high risk of choking should be made aware of these risks and receive choking prevention and first aid education. Given our positive experience with this choking prevention and education program, and ease of implementation, our methods may be generalizable to other clinicians in various clinical settings who see patient populations that, similarly, have an increased risk for choking.

We recognized several limitations of this QI project. Several caregivers declined to participate, and it is unknown how this could have impacted the results. Although it is known that one caregiver declined to participate due to prior training in CPR, it is possible that other caregivers who declined to participate also had prior training in CPR, as well. Likewise, it is unknown how many caregivers that did participate had prior training in CPR, although, anecdotally, we were aware of several. Many newborn infants with PWS require hospitalization in the neonatal intensive care unit (NICU) due to a poor respiratory effort and poor feeding, frequently requiring home respiratory support or gastrostomy tube al feeds at discharge. As such, it is common at our institution to offer basic CPR training to caregivers prior to the infants’ discharge home from the NICU. Although we did not systematically collect information regarding prior caregiver training in either those that declined or participated, it is possible that prior CPR training may have impacted the caregivers decision to participate, but also the caregivers response on both pre- and post-education surveys.

We also recognized the potential for recall and social bias. The post-education survey was administered to caregivers generally 10–15 min following the pre-education survey and immediately following the education session. It is possible that caregivers recalled their pre-education responses and sought to improve their responses on the post-education survey to demonstrate a gain of knowledge, or to satisfy the education facilitator or the medical team. Although surveys were administered to caregivers in a private setting without the education facilitator present to minimize these biases, it is likely that caregivers were aware of the medical team’s review of their response. In addition, we recognize that this QI project only assessed the effectiveness of our education program in the short-term and did not assess the long-term effectiveness of our education intervention. Although not a part of the initial QI project design, a follow-up survey to assess the long-term effect of the education program could be considered in a future iteration of this project to determine long-term effectiveness.

Finally, we recognized that the demographics of the PWS individuals in this QI project varied greatly, with PWS individuals ranging from infancy to adulthood, and with BMI and BMI z-scores ranging from severely underweight to very obese. This may have led to variation in the education provided to caregivers. The education facilitator would determine if the infant, child, or adult curriculum and mannequin was based on age, but also the general size of the PWS individual.

In conclusion, we found the implementation of a choking prevention and first aid education program into our PWS clinic to be an easy and effective strategy at improving caregiver awareness of choking risk in PWS individuals, self-reported knowledge of choking prevention strategies, and comfort in providing choking first aid. We suggest that the implementation of these preventative interventions and the choking education model can be implemented as standard practice in similar institutions and PWS clinics for all PWS caregivers.

## Figures and Tables

**Table 1:** Demographic information for PWS individuals cared for by caregivers.

Category	N = 45
Age (years)	
N	45
Mean (SD)	9.06 (6.53)
Range	0.25, 23.41
BMI, kg/m^2^	
Mean (SD)	22.95 (8.35)
Range	11.9, 49.44
BMI z-score	
Mean (SD)	1.03 (1.78)
Range	−3.29, 5.44
Age range, n (%)	
<2 yrs	6 (13%)
2–12 yrs	23 (51%)
>12 yrs	16 (36%)
Gender, n (%)	
Male	24 (53%)
Female	21 (46%)
Home environment, n (%)	
Two-caregiver household	35 (78%)
Single-caregiver household	9 (20%
Group home	1 (2%)

## Data Availability

All data generated or analyzed during this study are included in this published article.

## Supplementary Materials

The following are available online at https://www.mdpi.com/article/10.3390/jcm10214993/s1, File S1: Choking Prevention and First Aid Education in Prader-Willi Syndrome Pre/Post Test.
