# Supplementary material for: A Quality Improvement Project to Implement Choking Prevention and First Aid Education in Prader–Willi Syndrome Caregivers
Source: J Clin Med. 2021 Oct 27;10(21):4993. doi: 10.3390/jcm10214993 (PMC8584315; doi:10.3390/jcm10214993)
Supplement: Supplementary file 1 [file jcm-10-04993-s001.zip › jcm-1396886-supplementary.pdf]

NATIONWIDE CHILDREN'S
When your child needs a hospital, everything matters.℠

Choking Prevention and First Aid Education in Prader-Willi Syndrome

Pre/Post Test

1. How aware are you that individuals with Prader-Willi syndrome are at increased risk of choking?
   1-Not at all aware
   2-Somewhat aware
   3-Neutral
   4-Moderately Aware
   5-Very Aware

2. How informed/knowledgeable are you of ways to prevent choking for individuals with Prader-Willi syndrome?
   1-Not all informed/knowledgeable
   2-Somwhat informed/knowledgeable
   3-Neutral
   4-Moderately informed/knowledgeable
   5-Very informed/knowledgeable

3. How comfortable are you providing choking first aid?
   1-Not at all comfortable
   2-Somewhat comfortable
   3-Neutral
   4-Moderately comfortable
   5-Very comfortable
